# Sheep and Goat BSE Propagate More Efficiently than Cattle BSE in Human PrP Transgenic Mice

**DOI:** 10.1371/journal.ppat.1001319

**Published:** 2011-03-17

**Authors:** Danielle Padilla, Vincent Béringue, Juan Carlos Espinosa, Olivier Andreoletti, Emilie Jaumain, Fabienne Reine, Laetitia Herzog, Alfonso Gutierrez-Adan, Belen Pintado, Hubert Laude, Juan Maria Torres

**Affiliations:** 1 Centro de Investigación en Sanidad Animal (CISA-INIA), Madrid, Spain; 2 INRA, UR892, Virologie Immunologie Moléculaires, Jouy-en-Josas, France; 3 UMR INRA-ENVT 1225, Interactions Hôte Agent Pathogène, Ecole Nationale Vétérinaire de Toulouse, Toulouse, France; 4 Departamento de Reproducción Animal-INIA, Madrid, Spain; Istituto Superiore di Sanità, Italy

## Abstract

A new variant of Creutzfeldt Jacob Disease (vCJD) was identified in humans and linked to the consumption of Bovine Spongiform Encephalopathy (BSE)-infected meat products. Recycling of ruminant tissue in meat and bone meal (MBM) has been proposed as origin of the BSE epidemic. During this epidemic, sheep and goats have been exposed to BSE-contaminated MBM. It is well known that sheep can be experimentally infected with BSE and two field BSE-like cases have been reported in goats. In this work we evaluated the human susceptibility to small ruminants-passaged BSE prions by inoculating two different transgenic mouse lines expressing the methionine (Met) allele of human PrP at codon 129 (tg650 and tg340) with several sheep and goat BSE isolates and compared their transmission characteristics with those of cattle BSE. While the molecular and neuropathological transmission features were undistinguishable and similar to those obtained after transmission of vCJD in both transgenic mouse lines, sheep and goat BSE isolates showed higher transmission efficiency on serial passaging compared to cattle BSE. We found that this higher transmission efficiency was strongly influenced by the ovine PrP sequence, rather than by other host species-specific factors. Although extrapolation of results from prion transmission studies by using transgenic mice has to be done very carefully, especially when human susceptibility to prions is analyzed, our results clearly indicate that Met129 homozygous individuals might be susceptible to a sheep or goat BSE agent at a higher degree than to cattle BSE, and that these agents might transmit with molecular and neuropathological properties indistinguishable from those of vCJD. Our results suggest that the possibility of a small ruminant BSE prion as vCJD causal agent could not be ruled out, and that the risk for humans of a potential goat and/or sheep BSE agent should not be underestimated.

## Author Summary

Prion diseases, also referred as transmissible spongiform encephalopathies, are fatal neurodegenerative diseases caused by proteinaceous infectious particles denominated “prions.” Prion diseases acquired their first real public relevance with the outbreak of bovine spongiform encephalopathy (BSE) (“mad cow disease”) in the United Kingdom in the 80s and its link with the appearance of a new, variant form of Creutzfeldt-Jakob disease in humans. Recycling of ruminant tissues in meat and bone meal has been proposed as origin of the BSE epidemic. During this episode, sheep and goats have also been exposed to BSE-contaminated meal, so transmission to this species may have occurred. We analyzed the human susceptibility to sheep and goat passaged-BSE prions by using transgenic mice expressing human prion protein (PrP). When different sheep and goat BSE isolates were inoculated in these transgenic mice, higher susceptibility than that observed for cattle BSE was detected and the disease manifestation was similar to that observed in mice inoculated with the new variant of Creutzfeldt-Jakob disease. Our findings suggest that humans are at least equally, and might be even more, susceptible to a sheep or goat BSE agent compared to a cattle BSE one.

## Introduction

Transmissible Spongiform Encephalopathies (TSEs) are fatal neurodegenerative diseases which include Scrapie in sheep and goats, Bovine Spongiform Encephalopathy (BSE) and Creutzfeldt-Jakob disease (CJD) in humans. Prions, the causal agents of these diseases are thought to be infectious protein particles essentially composed of a misfolded isoform (PrP^Sc^) of the cellular prion protein (PrP^C^) [1], [2]. Scrapie has been detected more than two centuries ago, without epidemiological evidence of human transmission. BSE was diagnosed in cattle in the 80s [3] and subsequently acquired epidemic characteristics in several European countries. Ten years later, a variant form of CJD (vCJD) was identified in humans and linked to the consumption of BSE-infected products [4], [5]. During the BSE epidemic, sheep and goats have also been exposed to BSE-contaminated Meat and Bone Meal, so BSE transmission to these species may have occurred [6]. Sheep and goats are experimentally susceptible to BSE [7], [8], [9], [10], [11] and one confirmed [12] and one suspected [13] BSE-like case have been reported in goats in France and the United Kingdom (UK), respectively. While BSE infection is mostly restricted to the nervous system in cattle [14], [15], [16], [17], PrP^Sc^ is widely distributed in lymphoid tissues of experimentally BSE-infected sheep [18], [19], suggesting that infected sheep could provide a secondary and more dangerous source of BSE infection for humans.

Considering the protein-only hypothesis, one of the most difficult aspects to explain within prion diseases is the existence of prion strain diversity. Prion strains can be defined as isolates or sources of prion infectivity, that when transmitted to the same host, present distinct disease phenotypes, characterized by their incubation times, clinical signs, PrP^Sc^ biochemical properties, histopathological lesion profiles and PrP^Sc^ deposition patterns in the brain [20]. Intra-species prion transmission is characterized to be very efficient, maintaining these phenotypic traits on serial subpassaging. Although PrP primary sequence is highly conserved among mammals, inter-species prion propagation is limited by the so called transmission barrier, showing often at first passage lower attack rates and extended incubation times [21]. During years this transmission barrier has been called species barrier, suggesting to reside essentially in the degree of PrP amino acid sequence homology between donor and receiver [22], [23]. Nowadays there is strong evidence that also PrP^Sc^ conformation plays a critical role, not only in the cross-species transmission events, but also in the existence of different prion strains [24], [25], [26], [27], [28], [29].

In previous reports we demonstrated that BSE experimentally passaged in sheep homozygous for the A_136_R_154_Q_171_ allele of ovine PrP showed an enhanced virulence in transgenic mice expressing bovine and porcine PrP, compared to the original cattle BSE [29], [30]. The susceptibility of humans to a sheep or goat passaged BSE agent is still unknown. PrP polymorphism at codon 129, where either methionine or valine is encoded, has been described as a key factor influencing human prion susceptibility [31], [32], [33], [34] and seems to be particularly important in vCJD manifestation, as all but one clinical vCJD cases diagnosed so far are homozygous for methionine. In an attempt to evaluate the potential risk for humans of a sheep or goat BSE agent in this study we analyzed the susceptibility of transgenic mice expressing methionine 129 human PrP to sheep and goat BSE isolates.

## Results

In two parallel studies, performed in two different laboratories, the susceptibility of 2 lines of human-PrP transgenic mice to BSE agent was assessed without or after an intermediate passage in either sheep or goat. Both human-PrP transgenic mouse lines overexpress human M129 PrP under the mouse P*rnp* promoter on a mouse PrP null background but they differ in the genetic construction, genetic background and human PrP protein expression levels: a PAC insert, a Sv129 background and a 6-fold expression levels for tg650 mice [35] and a MoPrP.Xho vector, a 129Ola/B6CBA background and a 4-fold expression levels for tg340 mice (generated as described in Material and Methods). Tg650 (at INRA, France) and tg340 (at CISA-INIA, Spain) were inoculated with several cattle, sheep and goat BSE isolates (see Table 1 for a description of the isolates used). The transmission efficiency was evaluated by the appearance of TSE clinical symptoms and by the presence of PrP^res^ in the brain. The transmission data available to date, together with those obtained comparatively with vCJD or sCJD agents, are shown in Tables 2 and 3.

**Table 1:** Description of the different isolates used in this work.

Isolate	Origin(case number)	Description and references	Suplier
Ca-BSE_0_	Fr (139)	BSE naturally infected cow	INRA^1^
Ca-BSE_0_/TgBov		Pool of terminally ill bovine *tg110* transgenic mice inoculated with Ca-BSE_0_ [17], [30]	INIA^2^
Ca-BSE_2_	UK (PG1199/00)	BSE naturally infected cow [51], [56]	VLA^3^
Ca-BSE_2_/TgBov		Pool of terminally ill bovine Tg110 transgenic mice inoculated with Ca-BSE_2_ [51], [56]	INIA^2^
Ca-BSE_2_/TgOv		Pool of terminally ill ARQ/ARQ ovine TgshpIX transgenic mice [39] inoculated with Ca-BSE_2_	FLI^4^
Ca-BSE_3_	Fr (3)	BSE naturally infected cow [43]	INRA^1^
Ca-BSE_4_	Ge	BSE passaged in tgXV mice [43]	FLI^4^
Ca-BSE_5_	It (128204)	BSE naturally infected cow [43]	ISS^5^
Ca-BSE_6_	Be	BSE naturally infected cow [43]	LVTSEs^6^
Sh-BSE_0_	Fr (ARQ_0_)	Pool of terminally ill ARQ/ARQ sheep inoculated with Ca-BSE_0_ [17], [30]	INRA^1^
Sh-BSE_0_/TgBov		Pool of terminally ill bovine Tg110 transgenic mice inoculated with Sh-BSE_0_ [17]	INIA^2^
Sh-BSE_0_/TgOv		Pool of terminally ill ARQ/ARQ ovine TgshpIX transgenic mice [39] inoculated with Sh-BSE_1_	FLI^4^
Sh-BSE_2_	Fr (ARQ_2_)	ARQ/ARQ sheep inoculated with BSE [57]	AFSSA^7^
Sh-BSE_3_	Fr (ARQ_3_)	ARQ/ARQ sheep inoculated with BSE [57]	AFSSA^7^
Go-BSE_1_	Fr (CH636)	Goat BSE case [12]	AFSSA^7^
Go-BSE_1_/TgBov		2^nd^ passage of bovine tg540 mice inoculated with Go-BSE_1_	INRA^1^
Go-BSE_2_	UK	Goat experimentally infected with BSE	IAH^8^
vCJD_1_	UK (NHBY/0014)	vCJD M129M infected case [58]	NIBSC^9^
vCJD_2_	UK (NHBY0/0003)	vCJD M129M infected case	NIBSC^9^
vCJD_3_	Fr1	vCJD M129M infected case [35]	INSERM^10^
vCJD_4_	Fr2	vCJD M129M infected case [35]	INSERM^10^
vCJD_5_	Fr3	vCJD M129M infected case [35]	INSERM^10^
sCJD1	UK (NHBX0/0001)	Type I sCJD M129M infected case [58]	NIBSC^9^

1 French National Institute for Agricultural Research (INRA), Nouzilly, France.

2 Centro de Investigación en Sanidad Animal– Instituto Nacional de Investigación y Tecnología Agraria y Alimentaria (Madrid, Spain).

3 Veterinary Laboratory Agency (VLA), New Haw. Addlestone, Surrey, UK.

4 provided by Martin Groschup (Friedrich-Loeffler-Institut), Germany.

5 National Reference Centre for TSE (ISS Torino).

6 National Reference Laboratory for Veterinary TSEs (Belgium).

7 National TSE Reference Laboratory (AFSSA, Lyon, France).

8 provided by Nora Hunter (IAH, UK).

9 CJD Resource Centre-National Institute for Biological Standards and Control, South Mimms, Potters Bar, UK.

10 Institut National de la Santé et la Recherche Médicale (France).

**Table 2:** Transmission of bovine, ovine and goat-BSE isolates to tg650 (INRA).

Isolate	Mean survival time in days ± sem (n/n_0_)^a^			
	BoPrP-tg540 mice	HuPrP-tg650 mice		
	1^st^ passage	1^st^ passage	2^nd^ passage	3^rd^ passage
Ca-BSE_3_	298±7 (9/9)^b^	872 (1/6)^b^^,^ c	568±65 (6/7)^b^	527±14 (14/14)
Ca-BSE_3_	298±7 (9/9)^b^	627; 842 (2/6)^b^	677±54 (7/7)^b^	555±24 (8/8)
Ca-BSE_4_	269±11 (5/5)^b^	802 (1/4)^b^		
Ca-BSE_5_		606–775 (0/5)^b^		
Ca-BSE_6_	360±20 (6/6)^b^	696–829 (0/4)^b^		
Sh-BSE_2_	278±2 (6/6)^b^	749±50 (7/8)	596±21 (8/8)	
Sh-BSE_3_	339±5 (5/5)	581±60 (3/3)	462±21 (5/5)	518±11 (5/8)^d^
Go-BSE_1_	253±9 (6/6)^b^	571±67 (5/5)	597±16 (9/9)	534±12 (7/7)
Go-BSE_2_	343±98(5/5)	736±44 (6/6)		
vCJD_1_	343±8 (5/5)^b^	506±41 (6/10)^b^	491±37 (7/7)^b^	497±18 (10/10)^b^
vCJD_2_		518±11 (10/10)		
vCJD_3_		522±18 (5/5)^b^	520±26 (7/7)^b^	
vCJD_4_		512±15 (8/8)^b^		
vCJD_5_		515±41 (8/8)^b^		

a Intracerebral inoculation with 2 mg brain tissue equivalent; n/n_0_: diseased, PrP^res^: positive/inoculated animals.

b Data from Beringue *et al.* [35], [36].

c 1^st^ passage in hemizygous mice.

d Ongoing experiment (three animals still alive).

**Table 3:** Transmission of bovine, ovine and goat-BSE isolates to tg340 (CISA).

Isolates	Mean survival time in days ± sem (n/n_0_)^a^		
	BoPrP-tg110 mice	HuPrP-tg340 mice	
	1^st^ passage	1^st^ passage	2^nd^ passage
Ca-BSE_0_	303±10 (13/13)^b^	739 (1/6)	na
Ca-BSE_2_	308±5 (5/5)^b^	491–707 (0/9)	572±37 (3/4)
Sh-BSE_0_	234±5 (16/16)^b^	615±84 (4/6)	564±39 (5/5)
Go-BSE_1_	227±3 (7/7)	695±22 (6/7)	na
vCJD_1_	370±33 (9/9)	626±29 (6/6)	612±69 (6/6)
sCJD_1_		214±6 (5/5)	198±7 (6/6)

a Intracerebral inoculation with 2 mg brain tissue equivalent; n/n_0_: diseased, PrP^res^: positive/inoculated animals.

b Data from Espinosa *et al.* [29].

na, not available (experiments still ongoing).

### Low transmission efficiency of cattle-BSE prions in human-PrP mice

Both tg650 and tg340 lines were fairly susceptible to vCJD isolates with 100% clinical attack rates and mean survival times around 500 and 600 days post-infection (d.p.i.), respectively. These features were stable upon subpassaging, suggesting an absence of transmission barrier for this agent (Tables 2 and 3). Inoculation of cattle BSE isolates to tg340 mice produced markedly different results, as previously reported with tg650 mice [36]. At first passage, only one out of fifteen tg340 mice inoculated with BSE_2_ and BSE_0_ isolates was scored positive for brain PrP^res^ and at a very late stage (739 d.p.i.) without clear clinical signs. The remaining inoculated mice failed to develop a clinical disease or to accumulate detectable levels of PrP^res^ in the brain up to ∼700 days after inoculation. On second passage performed with brain homogenate from a PrP^res^-negative mouse (succumbed at 576 dpi) from the first passage (BSE_2_ isolate), 3 out of 4 inoculated tg340 mice tested positive for brain PrP^res^ by western blot with a survival time of 572±37 d.p.i. It is important to note that all the cattle BSE isolates tested in this study were transmitted as efficiently as vCJD isolates or other BSE-related sources to bovine PrP transgenic mice (Table 2 and 3), thus suggesting that they may have a comparable infectivity in the absence of an apparent transmission barrier [36]. Overall, these results indicate that both human-PrP transgenic mouse lines exhibit a strong transmission barrier to cattle BSE, suggesting that human PrP^C^ Met129 is a “bad substrate” for cattle BSE prions.

### High transmission efficiency of sheep and goat BSE isolates to human-PrP mice

We next examined the transmission efficiency of sheep- and goat-passaged BSE prions. Several experimental or “natural” isolates of distinct origin were selected (Table 1). Upon primary transmission to both tg340 and tg650 mouse lines, sheep and goat BSE isolates showed significant higher transmission ability than cattle BSE isolates. Thus the attack rates approached 100% (clinical signs, PrP^res^ detection in the brain) with almost all the sheep and goat BSE isolates used (Tables 2 and 3). Depending on the isolate used, the survival times varied between 571±67 and 749±50 d.p.i. in tg650 and 615±84 - 695±22 in tg340, thus much closer to the survival times observed for vCJD isolates inoculated in these mouse models (Tables 2 and 3). On second and third passage, 100% attack rates were obtained with all the sheep and goat BSE isolates tested. The incubation times were stable or for some isolates, slightly decreased and approached that of variant CJD. Overall, these data suggest a lower or an absence of apparent transmission barrier to sheep and goat BSE in human PrP transgenic mice.

**Figure 1:**
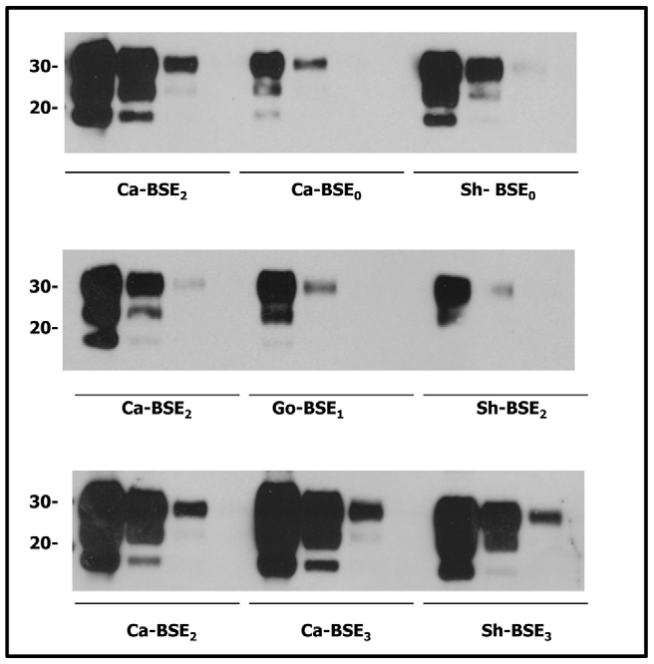
Comparative PrP^res^ content of the cattle, sheep and goat isolates used for tgHu bioassays. Immunoblots of brain PrP^res^ detected with SHA 31 mAb. Direct sample and 1/5 dilutions were loaded on 12% Bis-Tris gels. Molecular weights (in kDa) are shown at the left side of the blot. Excluding the BSE_0_ isolate, all cattle BSE isolates presented higher PrP^res^ contents than the sheep and goat BSE isolates. Cattle BSE_2_ and BSE_3_ isolates presented comparable PrP^res^ contents while that in the BSE_0_ isolate was substantially lower. The sheep and goat BSE isolates used contained similar PrP^res^ quantity.

Importantly, the comparatively higher attack rates seen with sheep and goat BSE isolates are not related to their initial PrP^res^ content as dilution experiment results indicate that cattle BSE isolates (Ca-BSE2 and Ca-BSE3) contained higher PrP^res^ levels in their brains than the sheep and goat BSE isolates (Figure 1).

### Human PrP transgenic mice accumulate vCJD-like PrP^res^ following inoculation with cattle, sheep or goat BSE

**Figure 2:**
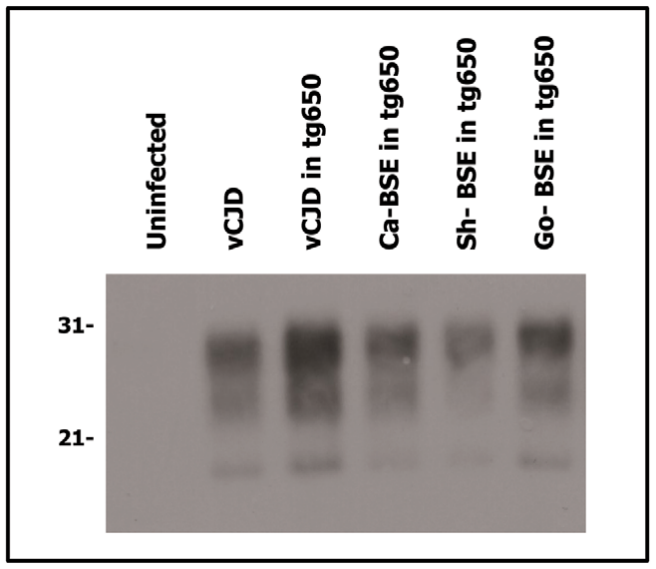
Western blots analysis of PrP^res^ in the brains of tg650 mice infected with human, bovine, ovine and goat isolates. Immunoblots of brain PrP^res^ detected with SHA 31 Mab. Similar amount of brain tissue equivalent (20 µg) was loaded for adequate comparison. vCJD isolate was loaded for comparison. Molecular weights (in kDa) are shown at the left side of the blot. Similar PrP^res^ banding patterns were observed in the vCJD- and the cattle, sheep and goat BSE-challenged tg650 mice.

**Figure 3:**
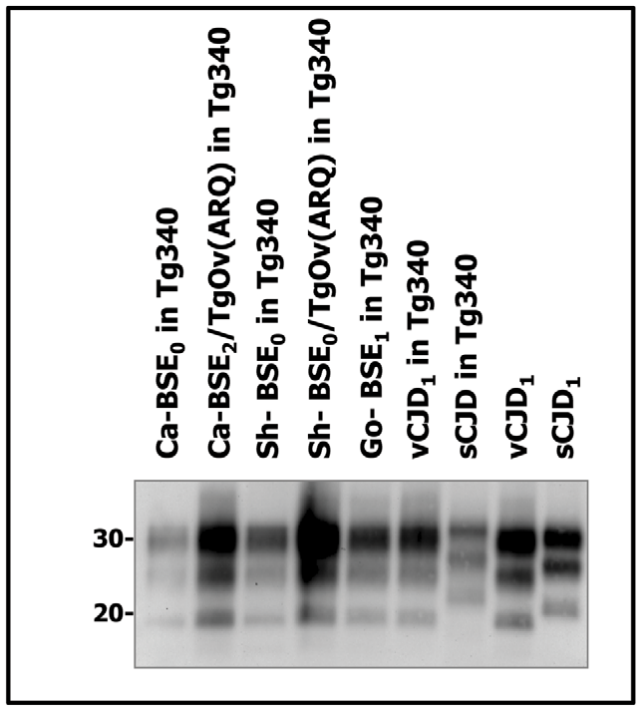
Western blots analysis of PrP^res^ in the brains of tg340 mice infected with human, bovine, ovine and goat isolates. Immunoblots of brain PrP^res^ detected with SHA 31 Mab. Similar quantities of PrP^res^ were loaded for adequate comparison. sCJD and vCJD_1_ isolates were loaded for comparison. Molecular weights (in kDa) are shown at the left side of the blot. Note the typical PrP^vCJD^ banding pattern, characterized by low size fragments and prominent diglycosylated species in the cattle, sheep and goat BSE-challenged mice. These banding patterns clearly differed from that observed after inoculation of mice with sporadic CJD.

**Figure 4:**
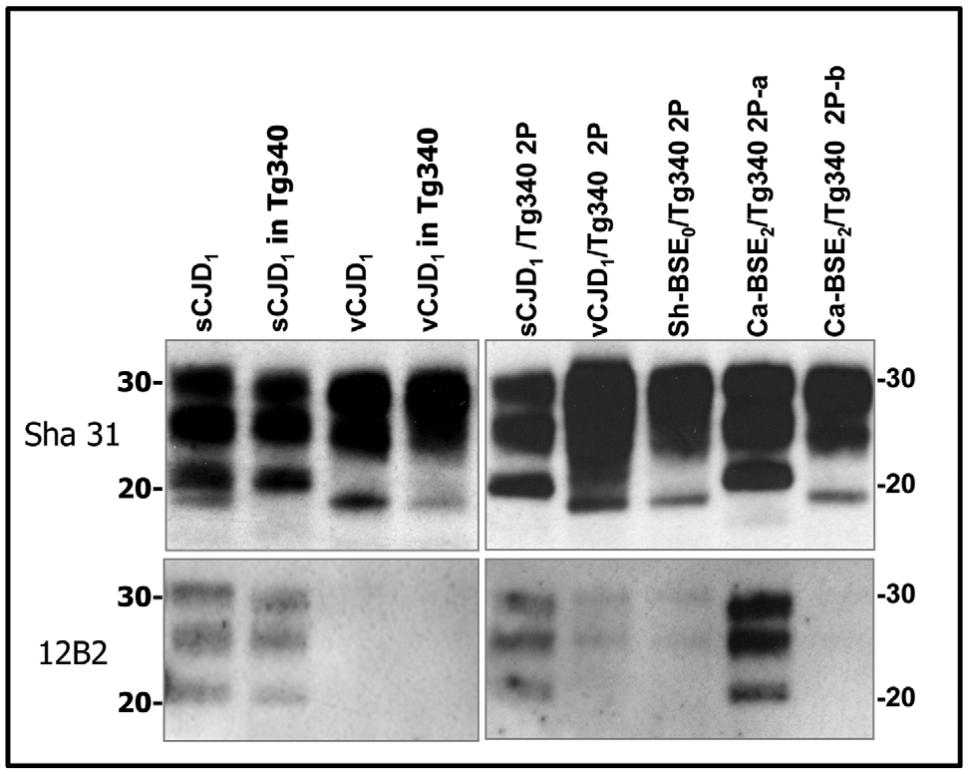
Western blots analysis of PrP^res^ in the brains of tg340 mice infected with human, bovine and ovine isolates. Immunoblots of brain PrP^res^ detected with SHA 31 and 12B2 mAbs. Similar quantities of PrP^res^ were loaded for adequate comparison. sCJD and vCJD_1_ isolates were loaded for comparison. Molecular weights (in kDa) are shown at the left side of the blot. Both vCJD- and type I sCJD-like PrP^res^ were observed after second passage of Ca-BSE_2_ in tg340 mice (Ca-BSE_2_/Tg340 2P-b and Ca-BSE_2_/Tg340 2P-a respectively).

Because distinct PrP^Sc^ conformations appear to encipher/encode distinct strains, brain PrP^res^ electrophoretic mobility and glycoprofile characterization constitutes standard criteria to distinguish between strains. Brain PrP^res^ of human PrP tg650 and tg340 transgenic mice inoculated with cattle, sheep and goat BSE isolates were analysed by western blot and the signature obtained was compared to that of variant CJD (Figures 2 and 3). A typical PrP^vCJD^ banding pattern, characterized by low size fragments (∼19 kDa fragment for the aglycosyl band) and prominent diglycosylated species was consistently observed in the challenged, PrP^res^-positive mice. This signature clearly differed from that observed after inoculation of mice with sporadic CJD (Figure 3 and [35]). Similar results were obtained when the immunoblots were performed with the 12B2 anti-PrP antibody, whose epitope (_89_WGQGG_93_ according to the human PrP sequence) is known to be poorly protected from proteinase K digestion [29], [37] in vCJD and BSE-related isolates (Figure 4). The only exception was observed after second passage of one cattle BSE isolate (Ca-BSE_2_) in tg340 mice, one of the three positive mice presented a brain PrP^res^ profile clearly distinct from PrP^vCJD^, that was comparable to that of typeI sCJD-inoculated tg340 mice with predominantly monoglycosylated and higher size fragments (∼21 kDa for the aglycosyl band) and preserved detection by 12B2 antibody (Figure 4).

### Cattle, sheep and goat BSE isolates showed similar neuropathological features in human PrP mice

**Figure 5:**
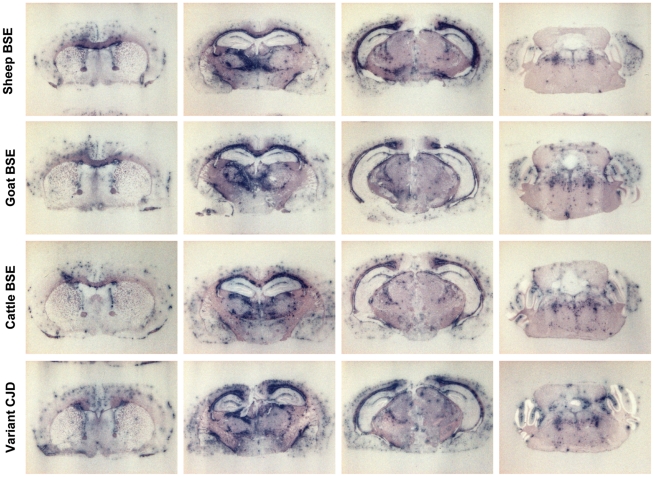
Regional distribution of PrP^res^ in the brain of human PrP transgenic mice infected with BSE agent passaged through sheep, goat or human species. Histoblots of 4 representative anteroposterior mouse brain sections are shown. Comparison has been made at 3^rd^ passage in tg650 mice. The patterns produced by sheep, goat and cattle BSE and variant CJD appear similar.

The regional distribution of PrP^res^ and vacuolation in the brains are standard criteria to differentiate between strains/TSE agents [38]. We thus compared the neuropathological phenotypes of cattle, sheep and goat BSE agents by PrP^res^ histoblotting and histopathological examination. The PrP^res^ deposition pattern of cattle, sheep and goat BSE were clearly similar in both tg650 and tg340 mice on 2^nd^ passage (not shown) and on 3^rd^ passage in tg650. As illustrated in Figure 5, large plaque-like PrP deposits were detected throughout the brain, predominantly in the cerebral cortex, corpus callosum, thalamic nuclei, optic tract, brain stem and cerebellum, a distribution which is similar to that seen in the brains of vCJD-infected tg650 mice (Figure 5 and [35]).

**Figure 6:**
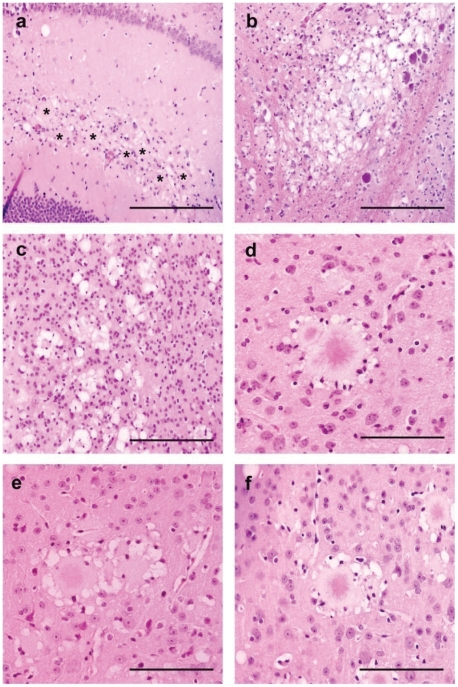
Brain vacuolar changes and amyloid plaques in transgenic mice for the human PRNP gene (methionine 129 variant - tg650). Hemalun-Eosin staining. Mice were inoculated with natural goat BSE (a – Bar 150 µm), cattle BSE (b: bar150 µm-e: bar 50 µm), vCJD (c: bar 150 µm-d: bar 50 µm) or experimental BSE in sheep (ARQ/ARQ genotype) (f: bar 50 µm). Vacuolar changes are associates to plaques deposits (a–b–c) (in a *: plaques deposits). Plaques deposits often harboured typical features of florid plaques: presence of crown of vacuoles and glial cell nuclei (d–e–f).

At microscopic level, abundant amyloid-like plaques were present (Figure 6), as suggested by histoblotting. These plaques were associated with severe vacuolisation of the surrounding tissue (‘florid like’ aspect see Figure 6), precluding the establishment of a reliable standard lesion profile. However similar distribution of the vacuolar changes was observed in the brain of mice inoculated with the different BSE and the vCJD isolates. It mainly involved thalamic, hippocampal and cerebral cortex areas, while brainstem and cerebellar cortex remained poorly affected (Figure 6).

### Transmission efficiency of BSE prions into human-PrP mouse models is strongly influenced by the agent PrP primary sequence

**Figure 7:**
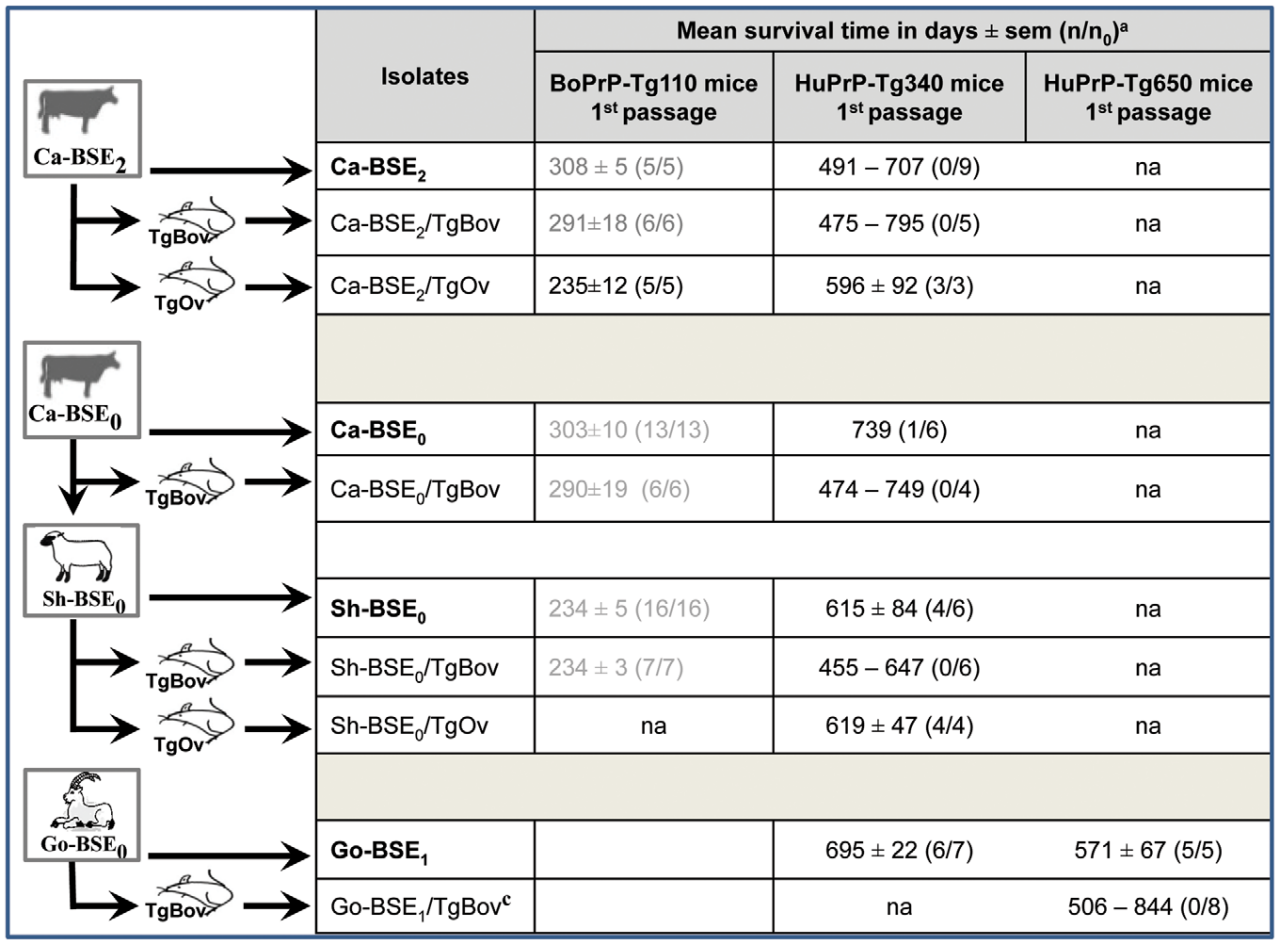
Transmission to human PrP mice of different BSE isolates generated by intermediary passages in either ovine or bovine PrP transgenic mice. na: not available (experiments still ongoing). ^a^Intracerebral inoculation with 2 mg brain tissue equivalent; n/n_0_: diseased, PrP^res^ positive/inoculated animals. ^c^Two serial passages on tgBov mice.

Once known that the phenotypes of cattle, sheep and goat BSE appear indistinguishable in human PrP mice, we proceed to analyze in more detail the potential elements involved in the change on BSE transmission characteristics after passage into sheep or goat. One of the cattle BSE isolate studied (Ca-BSE_2_) was passaged into bovine (*tg110*) and ovine PrP transgenic mice (ARQ allele) [39] to propagate BSE agents with different PrP primary sequence (these isolates were termed Ca-BSE_2_/TgBov and Ca-BSE_2_/TgOv, Table 1). The Ca-BSE_2_/TgBov isolate did not induce a clinical disease nor PrP^res^ accumulation in tg340 mice while an intermediate passage on the ovine PrP^ARQ^ sequence (Ca-BSE_2_/TgOv) restored the disease susceptibility, with survival times, biochemical and neuropathological features similar to those obtained with experimental sheep BSE isolates (Figure 7 and data not shown).

The opposite experiment was also performed. One sheep and one goat BSE isolate were passaged (twice for goat BSE) in bovine PrP transgenic mice (generating isolates Sh-BSE_0_/TgBov and Go-BSE_1_/TgBov, Table 1) before re-inoculation to human PrP transgenic mice (Figure 7). None of the mice inoculated with these isolates developed the disease nor accumulated PrP^res^, although one of them (Sh-BSE_0_/tgBov) produced the shortest disease in bovine PrP transgenic mice (Figure 7). This last result suggests that when sheep- or goat-BSE agents recovered their original bovine PrP sequence, the human transmission barrier was re-established. Moreover, the Sh-BSE_0_/TgOv isolate (which maintains its PrP ovine sequence) showed a full transmission rate in human transgenic mice with similar survival times as those of the original Sh-BSE_0_ isolate (Figure 7). Overall these data suggest that PrP^Sc^ primary sequence plays a critical role in the capacity of BSE prions to propagate on the human Met 129 PrP sequence.

## Discussion

In this study, we compared the transmission features of cattle and sheep/goat BSE prions in two different models of transgenic mice expressing Met129 human PrP (tg650 and tg340 lines) in two different laboratories. In general, the transmission results obtained in both human-PrP transgenic mouse lines were very comparable. Some shortening in survival times was observed in tg650 mice (compared to the tg340 mice line), which was probably due to higher PrP expression levels in this line. Worryingly, our results support the view that an intermediate passage of BSE agent in small ruminants accelerates the appearance of a vCJD-like disease in human PrP mice or markedly increases its transmission efficiency. Because the apparent phenotype of cattle and sheep/goat BSE prions is conserved, these data also unravel an important role of PrP^Sc^ primary sequence in the cross-species transmission capacities of prion strains.

The transmission efficiency of cattle BSE isolates in both human-PrP transgenic mouse models was apparently low. With all BSE isolates, whose high infectivity has been demonstrated in bovine-PrP transgenic mice (Tables 2 and 3), very low attack rates were obtained on primary transmission to both tg650 and tg340 mice. Three passages were necessary to achieve a degree of fitness comparable to vCJD in the same mouse line. This low BSE transmission efficiency to human PrP transgenic mice -occasionally accompanied by a strain shift- has also been described by others [40], [41], [42], and suggests a strong although not absolute transmission barrier. Although the exact characteristics and further evolution of the vCJD epidemic still entail uncertainties owing to prolonged incubation times, this apparent high transmission barrier of humans to cattle BSE might be an explanation for the currently low vCJD incidence, considering the high exposure to BSE during the “mad cow” crisis.

Remarkably, a different picture emerged when the sheep and goat BSE isolates were inoculated to human PrP transgenic mouse models. Attack rates approaching 100% were observed from the primary passage onwards and mean incubation times were more consistent with those measured after transmission of vCJD. On further passaging, the neuropathological phenotype and PrP^Sc^ type of cattle and sheep/goat BSE agents appeared indistinguishable from the vCJD agent propagated in these mice, as previously demonstrated in bovine transgenic mice [29], thus strongly supporting the view that the same BSE prion strain has been propagated whatever the infecting species. Hence, these observations reproduced in two distinct human transgenic lines with different genetic background and PrP expression levels support the view that transmission efficiency of BSE prions is increased by an intermediate passage in sheep or goat. Although the electrophoretic pattern of sheep/goat and cattle BSE PrP^res^ appeared similar in human-PrP transgenic mice, other assays are currently performed to further compare the biochemical or biophysical properties of the respective proteins are ongoing.

Importantly, the higher attack rates obtained after sheep and goat BSE transmissions compared to cattle BSE are not in accordance with the initial PrP^res^ content of these isolates. In addition, the data from inoculation to BoPrP-Tg reporter mice suggest that cattle BSE and sheep and goat-BSE isolates could have similar transmission efficiency (Table 1 and 2) in the absence of apparent transmission barrier [36]. Furthermore, when the human PrP transgenic lines were inoculated with the BSE agent passaged into bovine and ovine transgenic mice, the transmission results were comparable to those of the cattle and sheep BSE isolates (Figure 7), further supporting the crucial role of the PrP^Sc^ primary sequence in the increase of transmission efficiency. Taken together all these considerations suggest that the higher transmission efficiency of sheep and goat BSE isolates in comparison to cattle BSE isolates cannot be linked to a higher infectious titer of the inoculum but must be the outcome of a modification in the pathogenicity of the agent.

Commonly, transmission barriers are determined considering attack rates and quantified by measuring the fall in the mean survival times between the first and second passage. Hence, if we consider PrP^res^ detection as an indicator of successful transmission, our results imply that humans could be significantly more susceptible to a sheep or goat BSE agent than to a cattle BSE agent. On the other hand our results suggest that cattle BSE infection could produce very long latency in humans, with conversion efficiency far below the threshold of detectable PrP^res^, which is also very worrying since it suggests the possibility of silent carriers.

Our observations, made in two different mouse genetic backgrounds, suggest that the different transmission properties acquired by BSE after passage into either sheep or transgenic mice expressing ovine PrP are strongly related to the ovine PrP primary sequence, rather than to other host species-specific factors. Thus the transmission barrier observed with cattle BSE was fully restored when sheep/goat BSE experienced intermediate passaging into bovine transgenic mice before reinoculation to human PrP mice. In contrast, when the ovine sequence of sheep BSE was maintained, through passage into ARQ ovine PrP transgenic mice, the efficient transmission to human PrP mice was maintained. Apparently, an *ovine/caprine* PrP^Sc^ sequence appears to facilitate human PrP conversion by the BSE agent, compared to a bovine one.

The PrP primary sequence influence seems to depend strongly on the strain involved, since no PrP^res^ was found in either first or second passages of sheep scrapie in tg340 mice (unpublished observations), suggesting no infection, in accordance with the lack of epidemiological evidence linking scrapie with human TSE. Moreover, the low transmission efficiency observed for the cattle BSE agent is not exclusively linked to the bovine PrP sequence since other uncommon BSE strains (BSE-L) are efficiently transmitted to human-PrP mice [41], [43]. Considering the conformational selection model [20], our results would suggest that M129 human PrP^C^ prefers a BSE PrP^Sc^ with conformational characteristics templated by the ovine sequence, to a bovine BSE PrP^Sc^. Because a similar increased transmission efficiency of sheep/goat BSE has been reported in wild type mice [44] and transgenic mice expressing elk [45], bovine [29] and porcine [30] PrP, the better structural compatibility conferred by sheep/goat primary PrP^Sc^ sequence may not be limited to human PrP^C^. One explanation might be an alteration in the quaternary structure (after passage into sheep/goat) generating PrP^Sc^ polymers less degraded or more rapidly/easily amplified favouring or enhancing the initial conversion. This question is currently being addressed by sedimentation velocity [46] and PMCA experiments. Another possibility, within the quasispecies concept [20], [47], might be that BSE prions confrontation with the sheep and goat primary PrP sequence increases the variety of BSE substrain components, with the following emergence of a markedly adapted component in response to the selection pressure imposed by the interspecies transmission events. On the other hand, this component would not be distinguishable from bovine-passaged BSE prions due to the current limits of the standard biological methods and/or the molecular tools employed here to characterize prion strains. Whatever the mechanism, the notion that a passage through an intermediate species can profoundly alter prion virulence for the human species has important public-health issues, regarding emerging and/or expanding TSEs, like atypical scrapie or CWD.

Although extrapolation of results from prion transmission studies by using transgenic mice has to be done very carefully, especially when human susceptibility to prions is analyzed, our results clearly indicate that Met129 homozygous individuals might be susceptible to a sheep or goat BSE agent at a higher degree than to cattle BSE, and that these agents might transmit with molecular and neuropathological properties indistinguishable from those of vCJD. Although no vCJD cases have been described in Val129 homozygous individuals so far it is relevant to analyze if similar results will be observed in this genotype. This issue is currently being addressed in transmission experiments using transgenic mice expressing Val129 human PrP.

Taken all together, our results suggest that the possibility of a small ruminant BSE prion as vCJD causal agent could not be ruled out, which has important implications on public and animal health policies. On one hand, although the exact magnitude and characteristic of the vCJD epidemic is still unclear, its link with cattle BSE is supported by strong epidemiological ground and several experimental data. On the other hand, the molecular typing performed in our studies, indicates that the biochemical characteristics of the PrP^res^ detected in brains of our sheep and goat BSE-inoculated mice seem to be indistinguishable from that observed in vCJD. Considering the similarity in clinical manifestation of BSE- and scrapie-affected sheep [48], a masker effect of scrapie over BSE, as well as a potential adaptation of the BSE agent through subsequent passages, could not be ruled out. As BSE infected sheep PrP^Sc^ have been detected in many peripheral organs, small ruminant-passaged BSE prions might be a more widespread source of BSE infectivity compared to cattle [19], [49], [50]. This fact is even more worrying since our transmission studies suggest that apparently Met129 human PrP favours a BSE agent with ovine rather than a bovine sequence. Finally, it is evident that, although few natural cases have been described and so far we cannot draw any definitive conclusion about the origin of vCJD, we can not underestimate the risk of a potential goat and/or sheep BSE agent.

## Materials and Methods

### Ethics statement

Animal experiments were carried out in strict accordance with the recommendations in the guidelines of the Code for Methods and Welfare Considerations in Behavioural Research with Animals (Directive 86/609EC) and all efforts were made to minimize suffering. Experiments were approved by the Committee on the Ethics of Animal Experiments of the author's institutions (INRA and INIA); Permit Number: RTA06-091 and CT05-036353.

### TSE isolates

The isolates used in this study are described in Table 1. For mouse inoculation, all isolated were prepared from brain tissues as 10% (w/v) homogenates in 5% glucose.

### Mouse transmission studies

The tg650 transgenic mouse line over expresses human PrP M129 at a 6-fold level on a mouse PrP null background [35]. The tg340 mouse line expressing about 4-fold level of human PrP M129 on a mouse PrP null background has been generated following the same procedure previously described for the generation of other transgenic mouse line expressing different species PrP [51]. The details of this procedure are described below. Tg110 and tg540 mouse lines expresses bovine PrP at levels approximately 8-fold that in cattle brain [51], [52].

All inocula were prepared from brain tissues as 10% (w/v) homogenates. Individually identified 6–10 week-old mice were anesthetized and inoculated with 2 mg of brain homogenate in the right parietal lobe using a 25-gauge disposable hypodermic needle. Mice were observed daily and the neurological status was assessed weekly. When progression of a TSE disease was evident or at the end of lifespan, animals were euthanized because of ethical reasons. Once euthanized, necropsy was performed and brain was taken. A part of the brain was fixed by immersion in 10% formol to quantify spongiform degeneration by histopathology and PK resistant PrP accumulation (PrP^res^) by immunohistochemistry (IHQ) or histoblotting and the other was frozen at −20°C to determine presence of PrP^res^ by Western blot (WB). In all cases, survival time and attack rate were calculated for each isolate. Survival time was expressed as the mean of the survival days post inoculation (d.p.i.) of all the mice scored positive for PrP^res^, with its correspondent standard error. Attack rate was determined as the proportion of mice scored positive for PrP^res^ from all the mice inoculated. When all mice were scored negative for PrP^res^, the survival time range was shown. Brain homogenates from PrP^res^ positive mice, when available, were used for further passaging. When all mice were scored negative for PrP^res^ on primary passage, PrP^res^-negative brain homogenates were used for second passage.

### Western blot

175±20 mg of frozen brain tissue were homogenized in 5% glucose in distilled water in grinding tubes (Bio-Rad) adjusted to 10% (w/v) using a TeSeE™ Precess 48™ homogenizer (Bio-Rad) following manufacturer instructions. Presence of PrP^res^ in transgenic mice brains was determined by Western blot, following the procedure described below and using the reagents of the ELISA commercial test (TeSeE, Bio-Rad). 10–50 µl of a 10% (w/v) brain homogenate were diluted in a 10% (w/v) negative sheep brain homogenate, to obtain a 200 µl final volume. Homogenates were incubated for 10 min at 37°C with 200 µl of a 2% proteinase K solution (in buffer A). PrP^res^ was recovered as a pellet after addition of 200 µl of buffer B and a centrifugation at 15,000× *g* for 7 min at 20°C. Supernatants were discarded and pellets were dried inverted over absorbent paper for 5 min. Pellets were solubilised in Laemmli buffer and samples were incubated for 5 min at room temperature, solubilised, and heated at 100°C for 5 min. Samples were centrifuged at 20,000× *g* for 15 min at 20°C and supernatants were recovered and loaded on a 12% Bis-Tris Gel (Criterion XT, BioRad or NuPage, Invitrogen). Proteins were electrophoretically transferred onto PVDF or nitrocellulose membranes (Millipore). Membranes were blocked O/N with 2% BSA blocking buffer. For immunoblotting, membranes were incubated with either Sha 31 [53] or 12B2 [37] monoclonal antibody (Mab). Immunocomplexes were detected incubating the membranes for 1 hour with horseradish peroxidase conjugated anti mouse IgG (Amersham Pharmacia Biotech). Immunoblots were developed with enhanced chemiluminescence ECL Plus (GE Healthcare Amersham Biosciences).

### Histopathology

All procedures involving mice brains were performed as previously described [54]. Brain slices were realized, in order to allow lesion profiling according to the standard method described by Fraser and Dickinson [55]. Briefly, samples were fixed in neutral-1 buffered 10% formalin (4% 2- formaldehyde) before being cut at determined levels and paraffin embedded. After deparaffinization, 2 µm-thick tissue sections were stained with haematoxylin and eosin.

### Histoblots

Brains were rapidly removed from euthanised mice and frozen on dry ice. Thick 8–10 µm cryostat sections were cut, transferred onto Superfrost slides and kept at −20°C until use. Histoblot analyses were performed on 3 brains per infection at 2^nd^ and 3^rd^ passage, using the 3F4 anti-PrP antibody as previously described [35].

### Generation of tg340 mouse line expressing human PrP

Tg340 mouse line expressing about 4-fold level of human PrP M129 on a mouse PrP null background has been generated following a similar procedure previously describe for the generation of other transgenic mouse line expressing different species PrP [51], [56]. Briefly, the open reading frame (ORF) of human PrP gene was isolated by PCR amplification from human genomic DNA encoding methionine at codon 129. The primers used created a *XhoI* restriction enzyme site adjacent to the translation start and stop sites of the human PrP ORF (5′ -CTCGAGATTATGGCGAACCTTGGCTGCTGG- 3′ and 5′- CTCGAGTCATCCCACTATCAGGAAGATGAG- 3′, respectively). The PCR fragments obtained were sub cloned into a pGEM-T Easy Vector System (Promega) following manufacturer instructions, and inserts were sequenced to confirm no differences in the inferred amino acid sequence with respect to previously sequenced human PrP genes (GenBank accession number NM_183079) and to confirm the presence of the consequent codon 129 nucleotide variant (MetATG). The human PrP ORF was excised from the cloning vector using the restriction enzyme *XhoI* and inserted into the expression vector MoPrP.Xho [51], [56]. This vector contains the murine PrP promoter (including exon 1, intron 1, exon 2 and 3′ untranslated sequences) flanked by two *XhoI* restriction sites but could be distinguished from the wild type murine PrP gene because of the absence of intron 2. The vector was also digested with *XhoI* to excise the murine PrP ORF and the correspondent human PrP ORF were inserted by ligation, obtaining the plasmid pMo-huPrP129M.Xho.

The human transgene was excised from the plasmid vector using the restriction endonuclease *Not I* leading to DNA fragments of approximately 12 Kb. Finally, the DNAs were purified and dissolved in TE at a final concentration of 2 to 6 µg/ml and microinjected into pronuclear stage ova collected from super-ovulated B6CBAf1 females mated with 129/Ola males carrying a null mutation in endogenous PrP [51], [56].

DNA from founders' tails biopsies was extracted using a Extract-N-Amp Tissue PCR kit (Sigma-Aldrich) following manufacturer instructions. The presence of the human transgene in these founders was identified by PCR amplification using specific primers for the mouse PrP exon 2 and human PrP open reading frame. The absence of the murine PrP ORF in the transgenic mice generated was confirmed by PCR amplification using the primers: 5′- TAGATGTCAAGGACCTTCAGCC- 3′ and 5′- GTTCCACTGATTATGGGTACC -3′.

Eight different lines (founders) of human PrP^C^ (huPrP) and murine PrP^C^ (muPrP) heterozygous transgenic mice (PrP mu^+/−^ hu^+/−^) were obtained. The expression of human PrP^C^ in brain of these mouse lines was analyzed and compared with PrP^C^ content in human brain homogenate by western blot using mAb 3F4 which recognizes the _109_MKHM_112_ epitope (numbered according to the human PrP sequence). Human PrP^C^ was detected in 100% of the tested lines (data not shown). From the initial 8 different mouse lines heterozygous for both murine and human PrP genes (PrP mu^+/−^ hu^+/−^), the mouse line named as tg340 was selected for further experiments on the basis of the level of PrP^C^ expression.

**Figure 8:**
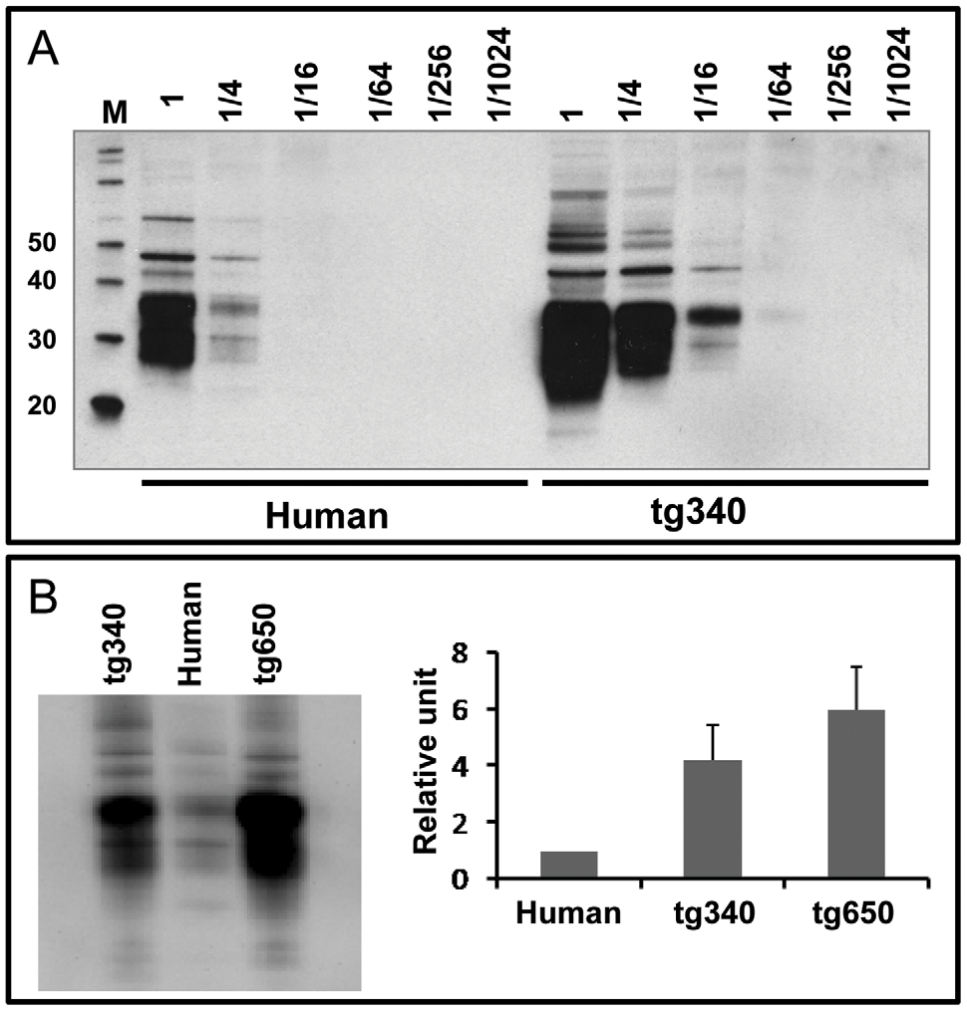
Brain PrP^C^ expression in homozygous tg340 mouse line in comparison to both tg650 mice and human brain. **A**) Immunoblots of the brain PrP^C^ expression in tg340 detected with Pri 308 Mab. Direct sample (10% brain homogenates) and ¼ dilutions were loaded on 12% Bis-Tris gels. The figure illustrates a representative set of three independent experiments. **B**) Brain PrP^C^ expression in tg340 in comparison to tg650 detected with Pri 308 Mab. Inmunoblots illustrates a representative set of three independent experiments and the diagrams represent the mean densytometric values data from these experiments. Data from human brains (*Human*) were considered as 1 relative unit. Error bars represent the standard deviation of the mean values.

Homozygous Tg340 mouse line was established backcrossing these animals with homozygous null animals MuPrP^−/−^ (*Prnp* ^−/−^) to obtain a null murine PrP background (PrP mu^−/−^ hu^+/−^). Interbreeding within these animals was performed to obtain homozygosis for the human PrP transgen within a murine PrP background (PrP mu^−/−^ hu^+/+^). The absence of murine PrP gene was determined by PCR using specific primers. Human PrP^C^ expression levels, determined more accurately in brain from homozygous tg340 animals was about 4-fold higher than PrP^C^ levels in human brain homogenates as determined by dilution experiments in western blot (Figure 8).

### Accession number

The GenBank accession number for the human *Prnp* gene used in this paper is NM_183079.
